# Nutritional Characterization, Antioxidant, and Lipid-Lowering Effects of Yellow Mombin (*Spondias mombin*) Supplemented to Rats Fed a High-Fat Diet

**DOI:** 10.3390/foods11193064

**Published:** 2022-10-02

**Authors:** Tatiana Luiza Costa Lucena, Kamila Sabino Batista, Rafael Oliveira Pinheiro, Hassler Clementino Cavalcante, Jéssyca Alencar de Sousa Gomes, Laiane Alves da Silva, Priscilla Paulo Lins, Fabrícia Souza Ferreira, Rafael Ferreira Lima, Marcos dos Santos Lima, Jailane de Souza Aquino

**Affiliations:** 1 Experimental Nutrition Laboratory—LANEX, Department of Nutrition, Federal University of Paraíba (UFPB), Campus I, João Pessoa 58051-900, Paraíba, Brazil; 2 Post Graduate Program in Nutrition Sciences, Federal University of Paraíba (UFPB), Campus I, João Pessoa 58051-900, Paraíba, Brazil; 3 Post Graduate Program in Food Science and Technology, Federal University of Paraíba (UFPB), Campus I, João Pessoa 58051-900, Paraíba, Brazil; 4 Postgraduate Program in Agroindustrial Systems, Campus Pombal, Federal University of Campina Grande (UFCG), Pombal 58840-000, Paraíba, Brazil; 5 Department of Food Technology, Federal Institute of Sertão Pernambucano (IF SertãoPE), Petrolina 56316-686, Pernambuco, Brazil

**Keywords:** adiposity, antioxidant enzymes, cholesterol, lipids, *Spondias mombin*

## Abstract

The aim of this study was to evaluate the effects of supplementing yellow mombin (YM) on the oxidative, somatic, and lipid parameters in rats fed a high-fat diet. A total of 24 adult Wistar rats were randomized into three groups: normal-fat diet (NF), high-fat diet (HF), and high-fat diet with YM supplementation (HFYM). Diets were administered for four weeks, and YM (400 mg/kg) was supplemented via gavage in the last two weeks of the experiment. After the four-week period, the somatic, serum biochemical, and liver oxidative parameters were evaluated. YM has a high antioxidant activity and significant amounts of phenolic compounds, carotenoids, vitamin C, dietary fibre, and minerals. The HFYM group had the lowest body weight (18.75%), body mass index (17.74%), and adiposity (31.63%) compared with the HF group. YM supplementation reduced low-density lipoprotein by 43.05% and increased high-density lipoprotein by 25.73%, but did not improve the triglyceride levels in the serum. YM treatment improved glucose tolerance and lipid peroxidation, and also enhanced the antioxidant capacity, superoxide dismutase, and glutathione peroxidase activities in the liver. These results indicate the lipid-lowering property and potential antioxidant activity of YM against liver oxidative damage caused by a high-fat diet intake, which may be associated with the bioactive compounds present in this fruit.

## 1. Introduction

The prevalence of cardiovascular diseases (CVDs) is considered to be a significant public health problem due to the high mortality rates worldwide [1]. Lifestyle, including food choices such as the exaggerated consumption of cholesterol, as well as saturated and trans fatty acids, at the expense of fibre consumption, are closely related to lipid metabolism disorders such as dyslipidaemias [2].

Dyslipidaemias are related to reduced levels of high-density lipoprotein (HDL) and/or the elevation of serum and/or tissue levels of triglycerides (TG), total cholesterol (TC), low-density lipoprotein (LDL), very low-density lipoprotein cholesterol (VLDL), and high-density lipoprotein (HDL), which, in turn, can contribute to the onset of non-alcoholic fatty liver disease, insulin resistance, and obesity (among other diseases) [3,4].

An unbalanced diet in the quantity and quality of fatty acids, and one that is rich in cholesterol can affect the antioxidant defence system, which can cause oxidative damage in various organs and tissues via the increased production of free radicals, especially in the liver, which is the main fat-metabolizing organ [5,6]. However, how the mechanisms involved in dysregulated human lipid metabolism alter antioxidant defence is still unclear. One of the hypotheses is that both high-fat diet fed mice and obese individuals would have a reduction in the expression of erythroid nuclear factor 2-related factor 2 (NRF2) in hepatic macrophages and hepatocytes, as the main role of NRF2 is to induce the expression of antioxidant response genes [7]. Metabolic diseases cause the depletion of superoxide dismutase (SOD), catalase (CAT), glutathione reductase, and glutathione peroxidase (GPx) activities, and also impair the non-enzymatic oxidative system, causing reduced thiol, vitamins, and minerals [8].

Despite decades of studies on lipid metabolism disorders and the development of various drugs such as statins, fibrates, nicotinic acid and variants, bile acid sequestrants, and cholesterol absorption inhibitors, their side effects remain under discussion [9,10]. On the other hand, dietary interventions such as reducing the consumption of foods rich in sugars, and saturated and trans fatty acids, as well as increasing the consumption of fruits and vegetables, have been shown to be effective in preventing and treating lipid metabolism disorders and CVD [11,12].

In this sense, studies have focused on the lipid-lowering and antioxidant potential of fruit pulp, pomace, and peels [13,14,15]. Yellow mombin (*Spondias mombin*) is among the tropical fruits produced and consumed in Brazil. It is part of the *Anacardiaceae* family and has a yellow-orange colour, smooth and thin skin, and a marked acid-sweet flavour. It is also known as cajá, taperebá, cajá-mirim, and cajá-verdadeiro [16]. Despite containing several bioactive compounds such as carotenoids, minerals, phenolic compounds, and fibres [17,18], this fruit is still underexplored, and few studies have addressed its functional potential to treat diseases [19,20]. Considering the aspects discussed above, the present study aimed to nutritionally characterize *Spondias mombin* and to evaluate the potential effects of its supplementation on th eoxidative, somatic, and lipid parameters of rats fed a high-fat diet.

## 2. Materials and Methods

### 2.1. Chemicals, Reagents, and Standards

Alpha-amylase, protease, and amyloglucosity enzymes; fructan; 1,1-diphenyl-2-picrylhydrazyl (DPPH); 2,2′-azino-bis (3-ethyl-benzothiazoline-6-sulfonic acid) (ABTS); ferric-reducing antioxidant power (FRAP) assay kit; 2,6-dichlorophenolindophenol reagent; and β-carotene were purchased from Sigma-Aldrich (Sigma-Aldrich, St. Louis, MO, USA). The external standards of organic acids, namely malic, lactic, tartaric, citric, butyric, propionic, formic, and acetic acids, and sugars, namely glucose, maltose, fructose, and rhamnose, were from Sigma-Aldrich (St. Louis, MO, USA). 

The external standards of the phenolics syringic acid, chlorogenic acid, gallic acid, p-coumaric acid, caffeic acid, trans-caftaric acid, 3,4-dihydroxybenzoic acid, 4-hydroxybenzoic acid, salycilic acid, vanillic acid, ellagic acid, hesperidin, naringenin, procyanidin B1, procyanidin B2, catechin, epicatechin and malvidin-3,5-diglucoside, and pelargonidin 3,5-diglucoside cyanidin 3,5-diglucoside were purchased from Sigma-Aldrich (St. Louis, MO, USA). Epicatechin, procyanidin A2, epicatechin gallate, epigallocatechin gallate, quercetin 3-glucoside, kaempferol 3-glucoside, rutin, myricetin, chrysin, delphinidin 3-glucoside, petunidin 3-glucoside, peonidin 3-glucoside, delphinidin 3-glucoside, cyanidin 3-glucoside, malvidin 3-glucoside, and pelargonidin 3-glucoside came from Extrasynthese (Genay, France). Trans-resveratrol and Cis-resveratrol were obtained from the Cayman Chemical Company (Ann Arbor, MI, USA).

### 2.2. Chemical Composition and Antioxidant Activity of Yellow Mombin

#### 2.2.1. Sample Preparation and Proximate Composition

Yellow mombin (*Spondias mombin* L.) from the city of Sapé, Paraíba, Brazil (Latitude: 07°05′47″ S, Longitude: 35°13′58″ W), was botanically identified and documented in the Lauro Pires Xavier Herbarium at the Federal University of Paraíba (UFPB). The fruits were selected according to the same stage of maturation (completely yellow skin colour), were sanitized in chlorinated water at a dilution of 200 ppm for 20 min, and were washed thoroughly. 

The pulp and peel were ground together, and the pits were discarded. Then, the samples were frozen and subjected to lyophilisation at −40 °C, vacuum pressure of 150 µHg, and lyophilisation at a speed of 1 m/h for approximately 12 h in a lyophiliser (model L-101, Liotop, Sao Carlos, SP, Brazil). The lyophilized sample was crushed in a food processor (Mixer Philips Walita, Itaipava, RJ, Brazil), sieved (1.0 mm mesh), then stored under freezing (−10 °C) and protected from light for further analysis [13].

All of the analyses to determine the chemical composition of yellow mombin (YM) were performed in triplicate. The proximate composition was determined through an analysis of the moisture by drying in an oven at 105 °C, ash by incineration in a muffle oven (550 °C), and proteins by the Kjeldahl method [21], in addition to the percentage of lipids using the Folch method [22]. The enzymatic-gravimetric method using alpha-amylase, protease, and amyloglucosity enzymes was used to quantify the total dietary fibre, insoluble dietary fibre, and soluble dietary fibre [21], and the fructan content was measured using enzymatic hydrolysis [21]. 

#### 2.2.2. Antioxidant Activity

Methanolic extract samples were first obtained by acid hydrolysis [23]. The in vitro antioxidant potential of YM was determined by ferric-reducing antioxidant power (FRAP) assay [24]; by 1,1-diphenyl-2-picrylhydrazyl (DPPH, Sigma-Aldrich, St. Louis, MO, USA) [25] and 2,2′-azino-bis (3-ethyl- benzothiazoline-6-sulfonic acid) (ABTS, Sigma-Aldrich, St. Louis, MO, USA) [26] radical scavenging assays; and with respective absorbance readings at 593 nm, 517 nm, and 734 nm using a spectrophotometer (Genesys 10S UV–VIS, Thermo Fisher Scientific, Madison, WI, USA). Data from the DPPH and ABTS assays were expressed as trolox equivalents (μmol TE/g) and data from the FRAP assay were expressed as μmol ferrous sulfate/g.

#### 2.2.3. Determination of Carotenoids and Ascorbic Acid

Ascorbic acid was determined using the colorimetric method with a 2,6-dichlorophenolindophenol reagent (Sigma-Aldrich, St. Louis, MO, USA) at 518 nm [27], and the quantification of carotenoids was performed at 450 nm using a calibration curve (0–50 ppm) prepared with β-carotene (Sigma-Aldrich, St. Louis, MO, USA) in hexane as the pattern [28]. Both analyses were performed using a spectrophotometer (Genesys 10S UV–VIS, Thermo Fisher Scientific, Madison, WI, USA).

#### 2.2.4. Sugars, Organic Acids and Phenolic Compounds

The monosaccharides, disaccharides, and organic acids of the YM were extracted with sulphuric acid at 4.0 mM/L [29]. The extracts (10 μL) were injected using a high-performance liquid chromatography instrument (HPLC; 1260 model, Agilent Technologies, Santa Clara, CA, USA) coupled to a refractive index detector (RID; model G1362A) and a diode array detector (DAD; model G1315D). Chromatographic separation was performed on an ion-exchange column, Agilent Hi-Plex H (300 × 7.7 mm, 8.0 µm) protected by a guard column Zorbax PL Hi-Plex H (5 × 3 mm) (Agilent Technologies, Santa Clara, CA, USA). The column temperature was maintained at 70 °C. The isocratic flow rate applied was 0.7 mL/min with a run time of 20 min. The phase was 4.0 mmol/L of H_2_SO_4_. Original external standards were injected to obtain the retention time for each compound. The detection of malic, lactic, tartaric, citric, butyric, propionic, formic, and acetic acids was conducted at 210 nm. In addition, detection for glucose, maltose, fructose, and rhamnose was carried out by RID. The calibration curves of the external standards were obtained (n = 5 points) using the least squares method. The identification and quantification of the compounds were done using the retention time of the sample peak in comparison with the retention time and the area of the peaks obtained in the external standards. All of the quantified compounds showed linearity with R2 > 0.995. The detection and quantification limits (LOD and LOQ, respectively) for all compounds evaluated were LOD < 0.042 g/L and LOQ < 0.131 g/L. Acids were detected at 210 nm and sugars were detected by RID [29]. 

The individual phenolic quantification of the YM was determined in methanolic extracts [30]. The extracts were filtered through a 0.45 μm membrane, dissolved in phase A (0.1 M phosphoric acid solution, pH = 2.0), and injected (20 μL). A Zorbax Eclipse Plus RP-C18 (100 × 4.6 mm, 3.5 µm) and pre-column Zorbax RP-C18 (12.6 × 4.6 mm, 5 µm) were used with the following analytical conditions [31]: solvent flow of 0.8 mL/min; the gradient used in the 0–5 min separation was 5% solvent B (methanol acidified with 0.5% H_3_PO_4_), 5–14 min (23% B), 14–30 min (50% B), and 30–33 min (80% B), at 35 °C. The detection was performed in DAD) at 220 nm for catechin, epicatechin, epicatechin gallate, epigallocatechin gallate, procyanidin B1, procyanidin A2, and procyanidin B2; 280 nm for gallic acid, syringic acid, salicylic acid, vanillic acid, hesperidin, cis-resveratrol, and naringenin; 320 nm for trans-caftaric acid, ρ-coumaric, chlorogenic acid, caffeic acid, ellagic acid, 3,4-dihydroxybenzoic acid, ellagic acid, 4-hydroxybenzoic acid, and trans-resveratrol; 360 nm for quercetin 3-O-glucoside, kaempferol 3-O-glucoside, rutin, myricetin, and chrysin; and 520 nm for malvidin 3-O-glucoside, cyanidin 3-O-glucoside, malvidin 3,5-diglucoside, cyanidin 3,5-diglucoside, pelargonidin 3-O-glucoside, delphinidin 3-O-glucoside, pelargonidin 3,5-diglucoside, and petunidin 3-O-glucoside. Calibration curves were obtained for five points and were related to the concentration versus peak area by regression analysis using the least squares method. The identification of the phenolics found in the present study was accomplished by comparing the retention time and UV spectrum of the sample peak with that obtained in the external standard. Quantification was performed by comparing the peak area in the sample with the calibration curve obtained using the external standards. All of the phenolics showed linearity (n = 5) with R2 > 0.997. The detection and quantification limits for all of the analysed compounds were LOD < 0.17 mg/L and LOQ < 0.38 mg/L.

Chromatographic analysis data were processed using the Open LAB CDS Chem Station Edition program (Agilent Technologies, Santa Clara, CA, USA). The chromatographic results were obtained in g/L of extract and were transformed to mg/g for organic acids and sugars, and µg/g for phenolic compounds. Flavonoid and non-flavonoid compounds were calculated from the sum of each compound, quantified by the chromatographic analysis.

#### 2.2.5. Mineral Profile

The mineral profile of the samples was analysed using an energy dispersive X-ray spectrometer device (model EDX-720, Tokyo, Japan) [32]. The YM samples were initially incinerated in a muffle oven at 550 °C, then placed in appropriate sample holders, sealed with a thin polypropylene film and a hole was opened at one end to prevent the extrusion of YM samples when activating the vacuum. 

### 2.3. Study Design, Diets, and Yellow Mombin Supplementation Administered to Wistar Rats

The biological assay was initiated after the protocol was approved by the Ethics Committee for the Use of Laboratory Animals of the Federal University of Paraíba (CEUA-UFPB), under protocol number 05052016, and followed the Animal Research guidelines: Reporting of In Vivo Experiments: the ARRIVE Guidelines [33].

**Figure 1:**
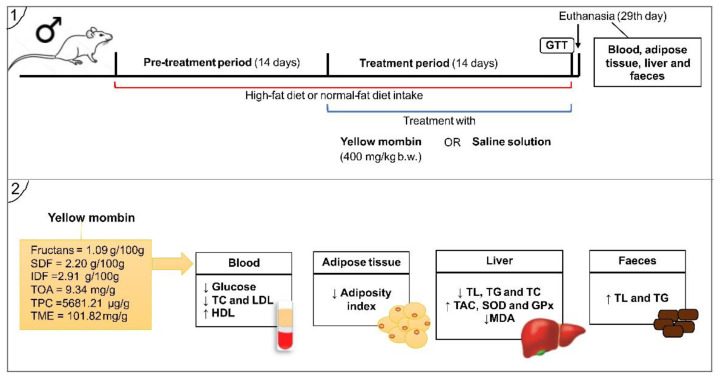
Experimental design. GTT = glucose tolerance test; HDL = high-density lipoprotein; IDF = insoluble dietary fibre; LDL = low-density lipoprotein; SDF = soluble dietary fibre; TC = total cholesterol; TAC = total antioxidant capacity; TG = triglycerides; TME = total mineral elements; TL = total lipids; TPC = total phenolic compounds; TOA = total organic acids.

A total of 24 male Wistar rats aged ± 80 days old were kept under standard lighting conditions (12/12 h light/dark cycle, lights off at 19:00 p.m.), humidity (55 ± 10%), and temperature (22 ± 2 °C). The rats were kept in collective polypropylene cages measuring 41 × 34 × 16 cm (four animals per cage), where they received filtered water and diets ad libitum. The rats were randomised into three groups: normal-fat diet (NF, n = 8), high-fat diet group (HF, n = 8), and high-fat diet group supplemented with yellow mombin (HFYM, n = 8) (Figure 1). 

The respective diets were administered for four weeks to the rats. The high-fat diet groups received a high-fat diet with 6% lard, 5% non-hydrolysed vegetable fat, 1% cholesterol, and 0.5% cholic acid (Rhoster, Araçoiaba da Serra, SP, Brazil), previously established as the period needed to dysregulate the serum lipid profile [13,34]. The normal-fat diet group (NF) consumed a maintenance diet (AIN-93M) proposed by the American Institute of Nutrition (AIN), during the same period, for which the lipid source was 4% soybean oil [35] (Supplementary Tables S1 and S2). 

YM was administered to YMHF rats in the last two weeks of the experiment at a dose of 400 mg/kg of body weight. The YM dose chosen (400 mg/kg) was established based on a pilot study, as well as on previous studies with the administration of lyophilised fruits, in which the effects against the damage induced by a high-fat diet in rodents were reported [13,15]. YM was diluted (1.6%, *w/v*) in saline and administered via a gavage twice a day with 4 h intervals in the morning and afternoon. The untreated groups (NF and HF) received a saline solution of the same volume during the last two weeks of the experiment [13].

### 2.4. Weight Gain and Food INTAKE Evaluation

The rats were weighed weekly (0, 7, 14, 21, and 28 days) on an electronic scale (Toledo Prixlll, São Bernardo do Campo, SP, Brazil). The food intake was measured weekly using the difference between the offered and remaining feed in grams [13].

### 2.5. Glucose Tolerance Test (GTT)

At the end of the four weeks of treatment, one day before euthanasia, the rats were submitted to 6 h of fasting before performing a glucose tolerance test (GTT). Blood was collected from the rats’ tails at 0 and 30, 60, and 90 min after gavage with a 25% glucose solution at a dose of 2 g glucose/kg body weight [34]. The blood glucose level of each animal was determined using a glucometer (Accu-check Performa, Jaguaré, SP, Brazil).

### 2.6. Euthanasia, Somatic Parameters, and Biological Material Collection

After the fourth week of treatment 48 h after completing the GTT, the rats were weighed on an electronic scale (brand Toledo model Prix III, São Bernardo do Campo, SP, Brazil) and were anaesthetised intraperitoneally (IP) with 75 mg/kg body weight of ketamine hydrochloride, associated with 10 mg/body weight kg of hydrochloride xylazine. Next, the body length (naso–anal), waist circumference (in the notch of the hind paws), and chest circumference (in the notch of the front paws) [36] were measured using an inelastic tape. The body weight (g) was divided by the squared length (cm²) to calculate the body mass index (BMI), and the Lee Index (LI) was calculated using the cube root of the body weight (g) divided by the length (cm).

Then, the rats were euthanised via cardiac puncture and aortic transection. The rats’ blood (4 mL) was collected and transferred to sterile tubes, which were centrifuged at 1040× *g*-force for 10 min to obtain the serum, which was kept at −20 °C until further lipid profiling. Then, the abdominal cavity was opened, and the liver, adipose tissue, and carcass were collected and weighed. The adiposity index was determined using the equation: (epididymal + visceral + retroperitoneal fat/final body weight) × 100, expressed in %) [37]. The liver (left lobe) was collected for the antioxidant parameter analysis.

### 2.7. Lipid Profile

The serum concentrations of the total cholesterol (TC), high-density lipoprotein cholesterol (HDL), low-density lipoprotein cholesterol (LDL), and triglycerides (TG) were measured using kits (Labtest^®^, Belo Horizonte, MG, Brazil), respectively. All of the analyses were performed on a LabMax 240 Premium automatic analyser (Labtest, Belo Horizonte, MG, Brazil), according to the manufacturer’s recommendations, at 500 nm (TC), 600 nm (HDL), 546 nm (LDL), or 505 nm (TG). The very low-density lipoprotein cholesterol (VLDL) quantity was calculated using TG/5.

### 2.8. Quantification of the Total Lipid, Cholesterol, and Triglyceride in the Faeces and in the Liver

The total lipid percentage in the faeces and liver was quantified by the cold extraction method [22]. Part of the lipid extracts was used to quantify TG and TC using specific commercial kits, as described in Section 2.6.

### 2.9. Liver Antioxidant Status

The antioxidant parameters were evaluated by analysing the lipid peroxidation, total antioxidant capacity (TAC), superoxide dismutase (SOD), and glutathione peroxidase (GPx) in the liver homogenates. First, each organ was ground and homogenised in 10 mL of potassium chloride (0.05 M) in an ice bath to prepare the homogenate. The homogenates were then centrifuged in a refrigerated centrifuge (Thermo Scientific Sorvall, st8, Waltham, MA, USA) (8000× *g* for 5 min at 4 °C), and the supernatant was retained for the analyses.

Lipid peroxidation was evaluated by measuring the thiobarbituric acid reactive substances (TBARS) and was expressed in terms of the malondialdehyde (MDA) content [38]. The reaction was analysed in an ultraviolet spectrophotometer (Bioespectro SP-22, Curitiba, PR, Brazil) at a wavelength of 532 nm at room temperature.

The TAC in the organ homogenates was measured by evaluating the free radical scavenging activity according to the DPPH method [25]. The reaction was analysed in an ultraviolet spectrophotometer (Bioespectro SP-22, Curitiba, PR, Brazil) at a wavelength of 515 nm and at room temperature.

The antioxidant activities of the superoxide dismutase enzymes (SOD, E.C.1.15. 1.1) and glutathione peroxidase (GPx; E.C. 1.11.1.9) were quantified in a hepatic homogenate using a RANSOD kit (Randox Laboratories Ltd., County Antrim, UK) and a RANSEL kit (Randox Laboratories Ltd., County Antrim, UK), respectively, conforming to the manufacturer’s instructions. Hepatic tissue was minced and homogenized in a 50 mM potassium phosphate buffer at pH 7.0 using a Potter-Elvehjem homogeniser to give a 10% (*w/v*) and centrifuged (8274× *g*/4 °C/4 min) product. The absorbance readings of 340 nm (GPx) and 505 nm (SOD) at 37 °C were performed in a Shimadzu 1650-PC UV–Visible spectrophotometer (Tokyo, Japan). The antioxidant activities were expressed in IU/mg protein for the liver.

### 2.10. Statistical Analysis

The sample size (24 rats randomized into three groups, n = 8 rats per group) was calculated to meet a minimum statistical power of 80%, with a minimally detectable effect size of 1.0 and a significance level of 0.05 (α = 0.05). The data and its residues were submitted to the Kolmogorov–Smirnov test and Levene’s test for the respective evaluation of the normal distribution and homogeneity of variance. Parametric data were analysed by analysis of variance (ANOVA) and Tukey’s post-test at a 5% significance level (*p* ≤ 0.05) using the SigmaPlot 12.5 version statistical package for evaluation (Systat Software Inc., San Jose, CA, USA). The MetaboAnalyst v.5.0 program (Xia Lab, McGill University, Montreal, Canada) was used for data pre-treatment with the auto scaling method and the creation of the correlation matrix. Pearson’s correlation coefficient (r) was used as a strength measure of the association between two variables (*p* ≤ 0.05).

## 3. Results and Discussion

### 3.1. Chemical Composition and Antioxidant Activity of Yellow Mombin

**Figure 2:**
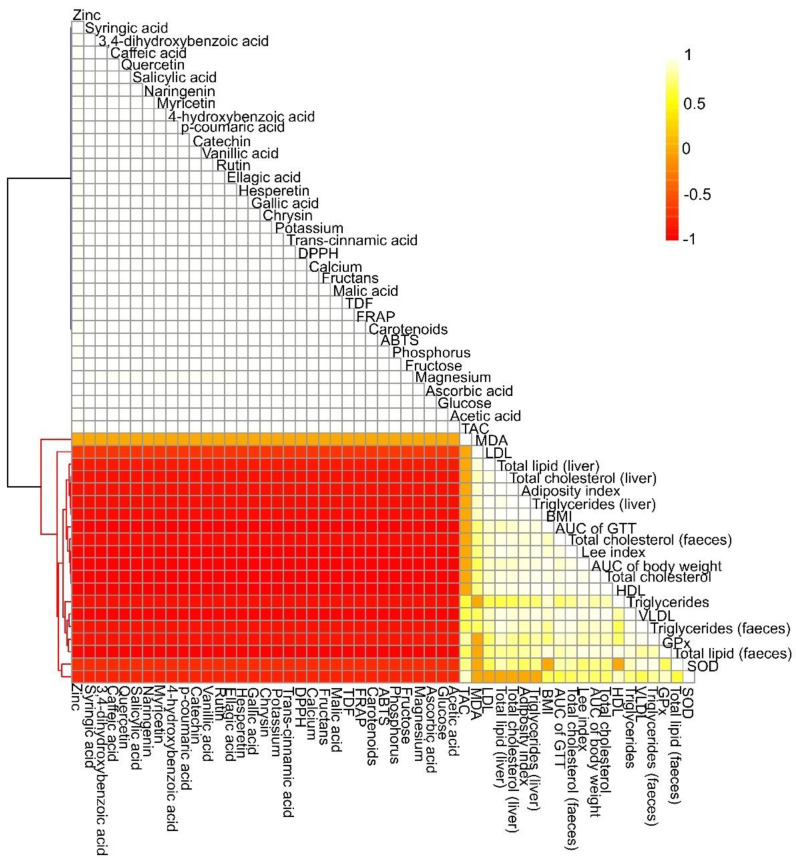
Hierarchical grouping based on Pearson’s correlation matrix between the chemical compounds of yellow mombin and the biological parameters of the rats fed a normal or high-fat diet and supplemented or not with yellow mombin. Significant correlations were determined based on r ≥ 0.6 and *p* ≤ 0.05. Positive and negative correlations are shown as yellow-white and red, respectively. Clustering of the correlation matrix reveals two clusters represented by colours: blue (compounds of yellow mombin) and red (biological parameters). ABTS = 2,2′-azino-bis (3-ethyl- benzothiazoline-6-sulfonic acid; AUC = area under the curve; BMI = body mass index; DPPH = 1,1-diphenyl-2-picrylhydrazyl; FRAP = ferric-reducing antioxidant power; GTT = glucose tolerance test; GPx = glutathione peroxidase; HDL = high-density lipoprotein; LDL = low-density lipoprotein; MDA = malondialdehyde; SOD = superoxide dismutase; TAC = total antioxidant capacity; TDF = total dietary fiber; VLDL = very low-density lipoprotein.

The YM characterization and antioxidant capacity are shown in Table 1. YM presented a variety of bioactive compounds in its composition, such as fructans, soluble dietary fibre, insoluble dietary fibre, malic acid, acetic acid, minerals, ascorbic acid, carotenoids, and phenolic compounds. Some of the major phenolic compounds found in YM are 3,4-dihydroxybenzoic acid, quercetin, caffeic acid, salicylic acid, vanillic acid, 4-hydroxybenzoic acid, rutin, and myricetin. All of the compounds quantified in the YM had a moderate to strong positive correlation (r = 0.06 to 1.00; *p* ≤ 0.001) with each other (Figure 2).

Our study demonstrated that YM has an important antioxidant activity, as well as large and varied amounts of bioactive compounds such as phenolic compounds, fibres, fructans, carotenoids, ascorbic acid, and minerals (Table 1). There is evidence that the bioactive compounds present in YM or fruits and their by-products of the same family (*Anacardiaceae*), such as cashews (*Anacardium occidentale*) and mangos (*Mangifera indica*), can be used in traditional and alternative medicine as an adjunct in the treatment of cardiovascular diseases [13,39]. 

In the present study, this evidence can be supported by a moderate to strong negative correlation (r = −0.06 to −1.00; *p* ≤ 0.001) between the compounds quantified in the YM and the biological analyses performed on rats (Figure 2). Herein, for the first time in the literature, we demonstrated that YM supplementation attenuated the changes induced by the high-fat diet in body weight, somatic parameters, glycaemia in GTT, lipid metabolism, and hepatic antioxidant status in the HFYM rats.

### 3.2. Effect on Weight Gain, Food, and Lipid Intake

**Figure 3:**
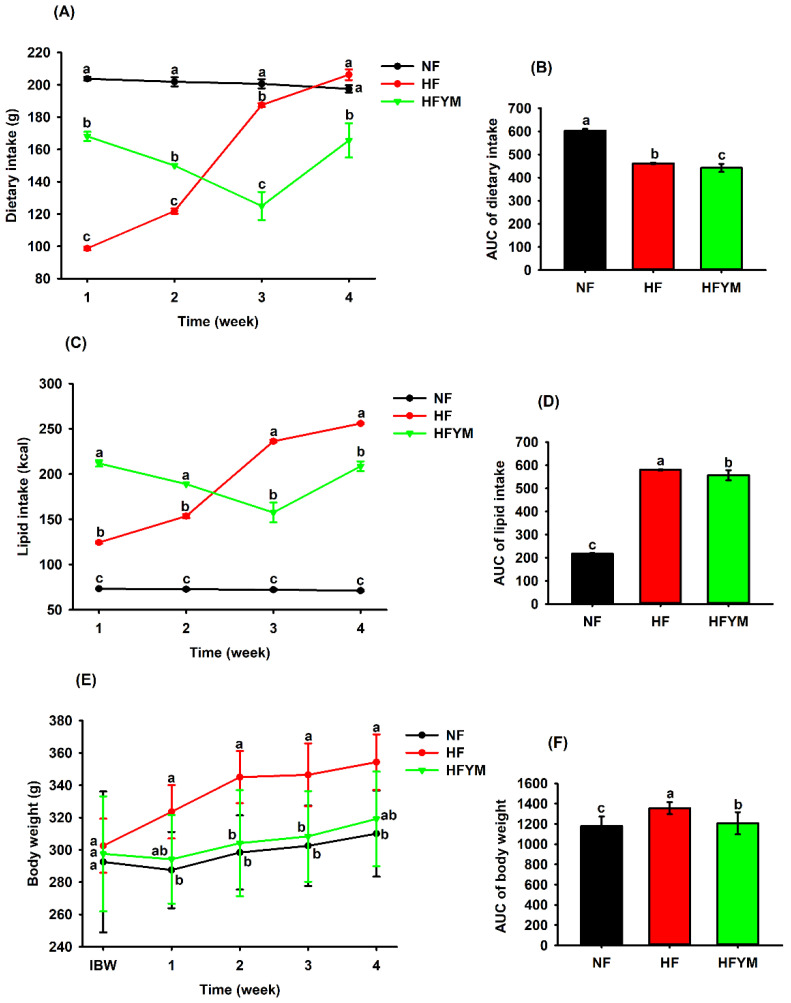
Dietary intake (**A**), lipid intake (**C**), body weight, (**E**) and respective areas under the curve—AUC (**B**,**D**,**F**). IBW = initial body weight; NF= normal-fat diet; HF= high-fat diet group; HFYM= high-fat diet group supplemented with yellow mombin. Data are represented with mean ± standard deviation. (a, b, c) Different lowercase letters at the same time or in the bars indicate that there was a statistical difference among the groups (one or two-way ANOVA followed by Tukey’s post-test, *p* ≤ 0.05).

The high-fat diet groups consumed the least amount of diet (Figure 3A,B) and more lipids (Figure 3C,D), which reflected in greater weight gain (Figure 3E,F) compared with the NF group (*p* ≤ 0.05). However, the HFYM group showed a lower dietary (Figure 3A,B) and lipid intake (Figure 3C,D) from the third week of the experiment, coinciding with the administration of YM, which resulted in less weight gain in this group (Figure 3E,F) when compared with the HF (*p* ≤ 0.05). In fact, previous studies have indicated that dietary fat consumption for a short time induces the secretion of satiety peptides in the gastrointestinal tract [13,40]. 

In addition, YM was able to reduce weight gain in rats fed a high-fat diet (HFYM) in comparison with the HF rats (Figure 3C). This effect may be associated with the combination of nutrients such as fat, fibre and polyphenols, which are present in the high-fat diet and YM, as they can potentiate the satietogenic effect. Fibres present in YM, especially insoluble ones, can prolong the contact of dietary fat with the intestinal mucosa and delay fat digestion, contributing to increased satiety [41]. Moreover, YM polyphenols can inhibit enzymes related to the digestion of carbohydrates and fats (amylase, glucosidase, and lipase), or even delay the secretion of appetite-stimulating hormones and inactivate appetite sensors [42].

### 3.3. Effect on Somatic Parameters

The group fed a high-fat diet (HF) had the highest carcass and liver weights, the highest abdominal and thoracic circumferences, and the highest adiposity index (*p* ≤ 0.05) among the groups, despite having a similar BMI and Lee index to the control group (*p* > 0.05). However, YM treatment was able to reduce all of the somatic parameters altered by the high-fat diet in the HFYM group, except for the Lee index (*p* ≤ 0.05) (Table 2). These results demonstrate that the Lee index may not be as sensitive as the BMI and adiposity index for rats fed this diet. Furthermore, it is important to highlight that despite the consumption of a high-calorie and high-fat diet, the rats in the HF and HFYM groups were not classified as obese, as they had a BMI below 0.68 g/cm^2^ [36], Lee index below 0.30 [43], and adiposity index below 6.3% for adult rats [37].

### 3.4. Effect of YM Supplementation on Glucose Tolerance

**Figure 4:**
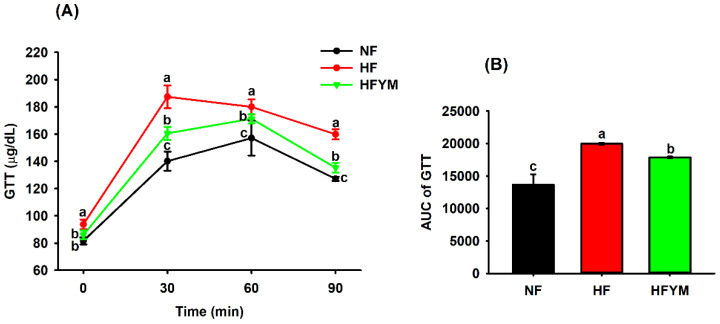
Glucose tolerance test (GTT) (**A**) performed in the rats fed a normal-fat or high-fat diet and supplemented or not with yellow mombin, as well as the area under the curve of (GTT AUC) of GTT (**B**). NF = normal-fat diet; HF = high-fat diet group; HFYM = high-fat diet group supplemented with yellow mombin. Data are represented with mean ± standard deviation. (a, b, c) = Different lowercase letters at the same time or in the bars indicate that there was a statistical difference among the groups (one way ANOVA followed by Tukey’s post-test, *p* ≤ 0.05).

Figure 4A shows a lower glucose tolerance in the HF group compared with the NF group from the initial time to the end of the test at 90 min. However, YM treatment reversed the glycaemic changes caused by the high-fat diet by reducing this parameter in the HFYM group compared with HF at all of the times analysed, as also demonstrated by the area under the curve (Figure 4B) (*p* ≤ 0.05). Previous studies have indicated that the compounds present in YM, such as phenolic compounds (flavonoids), soluble fibres, fructans, carotenoids, ascorbic acid, and some minerals (magnesium, potassium, and zinc), are involved in controlling glucose homeostasis and improving insulin sensitivity through different mechanisms. These compounds may have a synergistic action in glucose homeostasis [44,45,46,47,48,49], which can be supported by the fact of the area under the GTT curve was negatively correlated with the phenolic compounds (r = −0.91 to −0.92), minerals (r = −0.84 to −0.91), total dietary fibre, ascorbic acid, and carotenoids (r = −0.92, all) of YM (*p* ≤ 0.001) (Figure 2).

Caffeic acid is abundant in YM and acts in the suppression of hepatic glucose production through increasing glucokinase activity and glycogen production, reducing the hepatic activities of glucose 6-phosphatase (G6Pase) and phosphoenolpyruvate carboxy-kinase, and the declining glucose transporter 2 expression in hepatocytes [50].

This improvement in glycaemia in the GTT caused by YM supplementation (Figure 4) becomes particularly important, considering that most studies have evaluated the antidiabetic and hypoglycaemic effects of the extracts obtained from the leaves, roots, or seeds of the *Spondias genus*, and not from the edible part of the fruit, such as the pulp and peel [19,51].

### 3.5. Effect of YM Supplementation on the Serum Lipid Profile, Triglycerides, and Cholesterol Quantified in the Faeces and Liver

**Figure 5:**
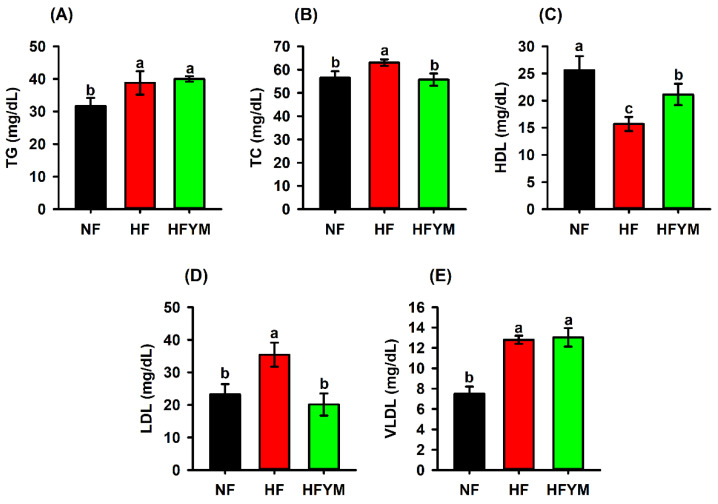
Triglycerides (**A**), total cholesterol (TC) (**B**), high-density lipoprotein HDL (**C**), low-density lipoprotein LDL (**D**) and very low-density lipoprotein VLDL (**E**) of rats fed a normal-fat or high-fat diet and supplemented or not with yellow mombin. TG = triglycerides; TC = total cholesterol; HDL = high-density lipoprotein; LDL = low-density lipoprotein; VLDL = very low-density lipoprotein; NF = normal-fat diet; HF = high-fat diet group; HFYM = high-fat diet group supplemented with yellow mombin. Data are represented with mean ± standard deviation. (a, b, c) Different lowercase letters in the bars indicate that there was a statistical difference among the groups (one way ANOVA followed by Tukey’s post-test, *p* ≤ 0.05).

The high-fat diet was able to increase the triglycerides (Figure 5A), total cholesterol (Figure 5B), LDL (Figure 5D), and VLDL levels (Figure 5E), in addition to reducing the HDL levels (Figure 5C) in relation to the control group fed a normal-fat diet (*p* ≤ 0.05). On the other hand, supplementation with YM reduced the total cholesterol (Figure 5B) and LDL levels (Figure 5D) and increased HDL levels (Figure 5C) in the HFYM group (*p* ≤ 0.0.5).

**Figure 6:**
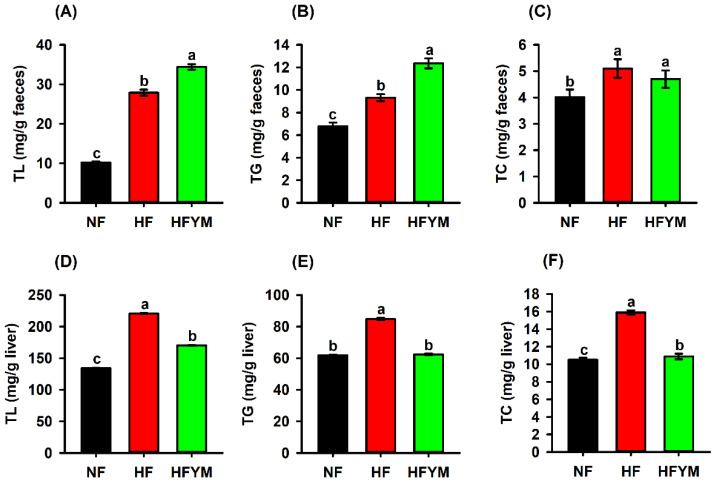
Total lipids (TL) (**A**,**D**), triglycerides (TG) (**B**,**E**), and total cholesterol (TC) (**C**,**F**) quantified in the faeces and liver of rats fed a normal-fat or high-fat diet and supplemented or not with yellow mombin. TL = total lipids; TG = triglycerides; TC = total cholesterol; NF = normal-fat diet; HF = high-fat diet group; HFYM = high-fat diet group supplemented with yellow mombin. Data are represented with mean ± standard deviation. (a, b, c) Different lowercase letters in the bars indicate that there was a statistical difference among the groups (one way ANOVA followed by Tukey’s post-test, *p* ≤ 0.05).

The HF group had a greater faecal excretion of total lipids (Figure 6A), triglycerides (Figure 6B), and total cholesterol (Figure 6C), as well as a greater lipid (Figure 6D), triglyceride (Figure 6E), and total cholesterol (Figure 6F) deposition in the liver in comparison with the control group (*p* ≤ 0.05). On the other hand, YM supplementation increased the faecal lipid (Figure 6A) and triglyceride (Figure 6B) excretion and reduced the hepatic lipid (Figure 6C), triglyceride (Figure 6E), and hepatic total cholesterol (Figure 6F) deposition in HFYM rats in comparison with the HF rats (*p* ≤ 0.05).

The increase in the faecal excretion of the total lipids and triglycerides in HFYM rats partially explains the improvement in the serum lipid profile and the reduced deposition of hepatic lipids (total, cholesterol, and triglycerides). On the other hand, the liver fat content reflects the balance between various metabolic pathways, such as lipolysis in the adipose tissue, lipogenesis, triglyceride esterification, fatty acid oxidation, and synthesis/secretion of lipoproteins in the liver tissue [52].

There are reports that the synergistic interaction of dietary fibre with flavonoids reduces the hepatic lipogenic enzyme activity, such as fatty acid synthase and HMG-CoA reductase, which are considered key enzymes in the fatty acid and cholesterol liver metabolism [53,54]. Furthermore, the positive and significant correlation between all of the compounds quantified in YM, as well as the negative correlation of dietary fibre (r = −0.88 to −0.99), phenolic compounds and minerals (r = −0.80 to −0.98), ascorbic acid (r = −0.85 to −0.99), and carotenoids (r = −0.85 to −0.99) from YM with serum lipids (TG, CT, LDL, and VLDL) (*p* ≤ 0.001; Figure 2) reinforce the hypothesis of the synergistic and potentially additive actions of the bioactive compounds quantified in YM on lipid metabolism. Quercetin is one of the major phenolic compounds in YM and is involved in regulating the LDL receptor gene expression, which can cause hypolipidaemic effects by increasing the clearance of circulating levels of LDL cholesterol from the blood [55]. The anti-hyperlipidaemic and hepatoprotective activities are also attributed to caffeic acid, which is one of the main phenolic compounds in YM and other fruits and vegetables [56].

### 3.6. Antioxidant Status in the Liver

**Figure 7:**
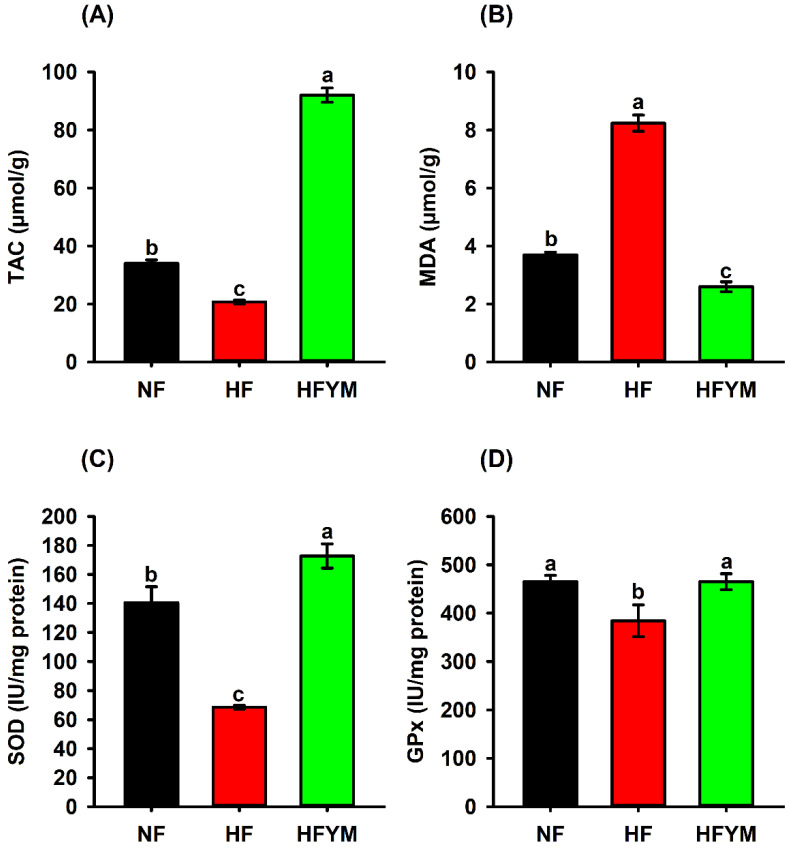
Total antioxidant capacity (TAC) (**A**), lipid peroxidation product—malondialdehyde—(MDA) (**B**), superoxide dismutase (SOD) (**C**), and glutathione peroxidase (GPx) (**D**) quantified in rat liver fed a normal-fat or high-fat diet and supplemented or not with yellow mombin. NF = normal-fat diet; HF = high-fat diet group; HFYM = high-fat diet group supplemented with yellow mombin. Data are represented with mean ± standard deviation. (a, b, c) Different lowercase letters in the bars indicate that there was a statistical difference among the groups (one way ANOVA followed by Tukey’s post-test, *p* ≤ 0.05).

The group fed a high-fat diet (HF) showed a reduced total antioxidant capacity (Figure 7A), increased lipid peroxidation (Figure 7B) and reduced antioxidant activity of SOD (Figure 7C) and GPx (Figure 7D) enzymes in the liver of rats compared with the control (*p* ≤ 0.05). On the other hand, YM supplementation was able to increase the total antioxidant capacity (Figure 7A) and SOD activity (Figure 7C), and also reduce liver lipid peroxidation (Figure 7B) in comparison with the control and HF groups, as well as increase the GPx activity (Figure 7D) compared with HF (*p* ≤ 0.05).

Lipid and glucose metabolisms are mainly regulated by the liver, and therefore we evaluated the antioxidant status in this organ. Lower MDA, higher TAC and activity of SOD and GPx in the liver of HFYM showed that YM increases antioxidant capacity and reduces oxidative degradation of lipids caused by continued consumption of the high-fat diet (Figure 7). Antioxidants such as ascorbic acid and carotenoids abundant in YM reinforce the defence against free radicals and non-radical oxidants, preventing the oxidative attack on cellular molecules (e.g., lipids, DNA and proteins) [57].

Interestingly, hepatic MDA showed a negative correlation with the antioxidant activity of YM determined by the FRAP, ABTS, and DPPH methods (r = −0.66, all; *p* ≤ 0.001), and hepatic MDA also showed a negative correlation (*p* ≤ 0.001) with the ascorbic acid (r = −0.66), carotenoids (r = −0.66), minerals (r = −0.60 to −0.66), and phenolic compounds (r = −0.66 to −0.67) quantified in YM (Figure 2). On the other hand, hepatic TAC had a positive correlation with hepatic SOD (r = 0.88) and GPx (r = 0.68) enzymes (*p* ≤ 0.001; Figure 2). These results highlight the importance of evaluating the biological activity of YM as a food matrix, considering the potential synergism between its components when faced with hepatic oxidative stress caused by the high-fat diet. Previous studies have reported that YM leaf supplementation improved antioxidant enzymes levels (CAT, SOD, and GPx) and lipid peroxidation (MDA) in the cardiac muscle of infarction-induced rats [20] and in the brains of rats induced to cyanide intoxication [58].

Although there are some differences in lipoprotein circulation and fat digestion between rats and humans [59], rats fed HFD have been a widely used animal model to evaluate the effects of different foods/herbal/nutraceuticals on lipid metabolism, liver health, and antioxidant status [13,15,34], as the synthesis of hepatic fatty acids and the transcriptional processes related to lipid metabolism are similar between these species [59].

Future studies to evaluate the effects of YM administration on enzyme activities and/or gene expressions related to lipid metabolism (e.g., acetyl CoA carboxylase, carnitine palmitoyl transferase 1, fatty acid synthase, and HMG-CoA reductase) and glycidic metabolism (e.g., glucokinase, G6Pase, glycogen synthase kinase-3, and phosphoenolpyruvate carboxykinase) are encouraged [60]. Furthermore, translational studies can be conducted to test the dose administered to rats, which is 64.86 mg/kg when converted to humans, with this calculation being based on equations that consider the body surface of these species [61].

## 4. Conclusions

The regular consumption of YM improved all of the somatic parameters of the rats, induced a blood glucose and cholesterol-lowering effect, reduced lipid accumulation in the liver, increased faecal lipid excretion, and protected the liver from oxidative damage caused by the consumption of a high-fat diet.

Our results suggest that the effects of YM on the lipid metabolism and antioxidant defence disorders caused by a high-fat diet are associated with the action of dietary fibre, fructans, carotenoids, ascorbic acid, some mineral elements, and phenolic compound contents quantified in this food matrix, which may direct future studies to uncover evidence of the functional/nutraceutical potential of this fruit.

## Figures and Tables

**Table 1:** The chemical composition and antioxidant activity of yellow mombin.

Parameters (Dry Basis)	Yellow Mombin	Parameters (Dry Basis)	Yellow Mombin
Ash (g/100g)	5.21 ± 0.06	**Phenolics compounds**	
Moisture (g/100g)	13.51 ± 0.20	**Hydroxybenzoic acids (µg/g)**	
Protein (g/100g)	7.12 ± 0.10	Gallic acid	16.02 ± 0.02
Lipid (g/100g)	1.43 ± 0.25	Syringic acid	80.18 ± 0.02
Glucose (mg/g)	0.16 ± 0.01	Salicylic acid	618.89 ± 0.12
Fructose (mg/g)	0.17 ± 0.02	Vanillic acid	528.78 ± 0.26
Fructans (g/100g)	0.99 ± 0.02	**Hydroxycinnamic acids (µg/g)**	
SDF (g/100g)	6.20 ± 0.04	Trans-cinnamic acid	2.23 ± 0.02
IDF(g/100g)	8.91 ± 0.07	Caffeic acid	764.41 ± 0.21
TDF(g/100g)	15.11 ± 0.11	p-coumaric acid	216.56 ± 0.09
IDF:SDF ratio	1.44	* Sum of non-flavonoids (µg/g)	**4153.59**
**Organic acids (mg/g)**		**Mineral elements (mg/g)**	
Acetic acid	3.34 ± 0.21	Potassium	83.06 ± 0.12
Malic acid	6.00 ± 0.12	Calcium	9.35 ± 0.11
**Phenolics compounds**		Chlorine	3.53 ± 0.07
**Flavanol (µg/g)**		Phosphorus	2.65 ± 0.08
Catechin	38.18 ± 0.02	Magnesium	1.82 ± 0.17
**Flavanone (µg/g)**		Sulphur	1.06 ± 0.03
Hesperetin	28.02 ± 0.03	Iron	0.22 ± 0.02
Naringenin	28.05 ± 0.02	Calcium	0.12 ± 0.02
Chrysin	12.04 ± 0.02	Copper	0.06 ± <0.01
**Flavonols (µg/g)**		Zinc	0.05 ± <0.01
Myricetin	322.01 ± 0.19	Cobalt	0.02 ± <0.01
Quercetin	774.09 ± 0.10	**Other antioxidant compounds (mg/100 g)**	
Rutin	325.29 ± 0.15	Ascorbic acid	238.06 ± 15.60
* Sum of flavonoids (µg/g)	**1527.62**	Carotenoids	17.81 ± 0.37
**Hydroxybenzoic acids (µg/g)**		**Antioxidant activity**	
3,4-dihydroxybenzoic acid	1390.20 ± 0.40	DPPH (µmol TE /g)	669.61 ± 7.90
4-hydroxybenzoic acid	420.65 ± 0.17	ABTS (µmol TE /g)	498.76 ± 16.14
Ellagic acid	105.67 ± 0.05	FRAP (μmol ferrous sulfate/g)	1205.24 ± 25.17

Data are represented with mean ± standard deviation. ABTS = 2,2′-azino-bis (3-ethyl- benzothiazoline-6-sulfonic acid; DPPH = 1,1-diphenyl-2-picrylhydrazyl; FRAP = ferric-reducing antioxidant power; IDF= insoluble dietary fibre; SDF = soluble dietary fibre; TE= trolox equivalent; TDF= total dietary fibre. * Sum of the amounts of phenolic compounds per group (flavonoids and non-flavonoids) obtained by chromatographic analysis.

**Table 2:** Somatic parameters of rats fed a normal-fed or high-fat diet and supplemented or not with yellow mombin.

Somatic Parameters	NF	HF	HFYM
Abdominal circumference (cm)	16.10 ± 0.42 b	17.29 ± 0.76 a	15.07 ± 0.45 b
Thoracic circumference (cm)	14.28 ± 0.68 b	15.13 ± 0.35 a	13.75 ± 0.60 b
Final body weight (g)	304.00 ± 24.85 a	330.00 ± 15.81 a	268.13 ± 20.86 b
Body length (cm)	22.81 ± 0.75 a	23.44 ± 0.78 a	22.94 ± 0.90 a
BMI (g/cm^2^)	0.62 ± 0.06 a	0.62 ± 0.03 a	0.51 ± 0.03 b
Lee index	0.24 ± 0.01 a	0.24 ± 0.03 a	0.23 ± 0.04 a
Adiposity index (%)	2.82 ± 0.64 b	3.70 ± 0.15 a	2.53 ± 0.28 b
Carcass weight (g)	184.17 ± 24.38 b	207.50 ± 9.35 a	163.14 ± 17.39 b
Liver weight (g)	8.33 ± 0.89 b	10.25 ± 0.91 a	8.63 ± 0.49 b

BMI = body mass index; NF = normal-fat diet; HF = high-fat diet group; HFYM = high-fat diet group supplemented with yellow mombin. Data are represented with mean ± standard deviation. (a, b, c) Different lowercase letters in the same row indicate that there was a statistical difference among the groups (one way ANOVA followed by Tukey’s post-test, *p* ≤ 0.05).

## Data Availability

Data is contained within the article or Supplementary Material.

## Supplementary Materials

The following supporting information can be downloaded at: https://www.mdpi.com/article/10.3390/foods11193064/s1, Table S1. Composition of the normal-fat and high-fat diets consumed by Wistar rats treated or not with yellow mombin. Table S2. Fatty acid composition of the normal-fat and high-fat diets consumed by Wistar rats treated or not with yellow mombin.
