# Supplementary material for: Nutritional Characterization, Antioxidant, and Lipid-Lowering Effects of Yellow Mombin (Spondias mombin) Supplemented to Rats Fed a High-Fat Diet
Source: Foods. 2022 Oct 2;11(19):3064. doi: 10.3390/foods11193064 (PMC9563763; doi:10.3390/foods11193064)
Supplement: Supplementary file 1 [file foods-11-03064-s001.zip › foods-1915299-supplementary.pdf]

Supplementary material

Table S1. Composition of the normal-fat and high-fat diets consumed by Wistar rats treated or not with yellow mombin.

**Ingredients (g/100g)**	**NF**	**HF**
Milk casein	14.00	16.50
Maize starch	47.00	36.45
Dextrinized starch	15.50	15.50
Sucrose	10.00	6.00
Fibre	5.00	5.00
Soybean oil	4.00	3.00
Lard (animal fat)	-	6.00
Non-hydrolyzed vegetable fat	-	5.00
Cholic acid	-	0.50
Cholesterol	-	1.00
Mineral mix AIN -93M	3.50	3.50
Vitamin mix	1.00	1,00
L-cysteine	0.18	0.30
Hill bitartrate	0.25	0.25
t-BHQ	0.008	0.014
Energy value (kcal/g)	3.82	4.25
Carbohydrates (kcal %)	75.77	54.54
Proteins (kcal %)	14.82	15.81
Lipids (kcal %)	9.40	29.64

NF= normal fat-diet/AIN-93M diet maintenance proposed by American Institute of Nutrition (AIN) [35]; HF= high-fat diet (Rhoster Industria and Comércio Ltda); t-BHQ= tert-butylhydroquinone.

Table S2. Fatty acid composition of the normal-fat and high-fat diets consumed by Wistar rats treated or not with yellow mombin.

Fatty Acids (FA) (g/100g)	NF	HF
Decanoic acid C10:0	0.02± <0.01	0.05± <0.01
Dodecanoic acid C12:0	-	0.09±<0.01
Tetradecanoic acid C14:0	0.13± 0.01	1.01±0.02
Pentadecanoic acid C15:0	-	0.05±<0.01
Hexadecanoic acid C16:0	12.83± 0.08	28.65±0.18
Heptadecanoic acid C17:0	0.08± <0.01	0.34±<0.01
Stearate acid C18:0	3.80±0.05	17.21±0.05
Eicosanoic acid C20:0	0.33±0.04	0.50±<0.01
Docosanoic acid C22:0	0.38±0.05	0.37±<0.01
**Saturated Fatty Acids (SFA)**	**17.62**	**48.34**
9-Hexadecenoic acid C16:1 ω7	-	1.72±<0.01
Cis-10-Heptadecenoic acid C17:1	-	0.10±<0.01
11-Octadecenoic acid C18:1 ω7	-	0.94±0.06
cis-13-Octadecenoic acid C18:1	-	0.82 ±0.02
Octadecenoic acid C18:1 ω 9	22.46±0.08	6.78±0.06
9-Octadecenoic acid C18:1 ω9t	-	0.18±0.02
cis-13-Eicosenoic acid C20:1 ω7	-	0.62±0.01
**Monounsaturated Fatty Acids (MUFA)**	**22.46**	**11.16**
9,12-Octadecadienoic acid C18:2 ω6c	53.88±0.04	37.02 ±0.32
9,12,15-Octadecatrienoic acid C18:3 ω3	6.04±0.06	2.83 ±0.01
cis-11,14-Eicosadienoic acid C20:2 ω6	-	0.46±0.04
5,8,11,14-Eicosatetraenoic acid C20:4 ω6	-	0.18±0.01
**Polyunsaturated Fatty Acids (PUFA)**	**59.90**	**40.50**

NF= normal fat-diet/AIN-93M diet maintenance proposed by American Institute of Nutrition (AIN) [35]; HF= high-fat diet (Rhoster Industria and Comércio Ltda).
